# Effects of a caffeine-based diet on insecticide resistance and longevity in infected *Anopheles coluzzii*

**DOI:** 10.1186/s13071-026-07253-z

**Published:** 2026-02-13

**Authors:** Khadidiatou Cissé-Niambélé, Jacob C. Koella, Domonbabele François de Sales Hien, Benjamin Guibéhi Koudou

**Affiliations:** 1 Institute of Biology, University of Neuchâtel, Neuchâtel, Switzerland; 2 Centre Suisse de Recherches Scientifiques en Côte d’Ivoire, Abidjan, Côte d’Ivoire; 3 UFR Sciences de La Nature, Laboratoire de Cytologie Et Biologie Animales, Université Nangui Abrogoua, Abidjan, Côte d’Ivoire; 4 Institut de Recherche en Sciences de La Santé (IRSS), Laboratoire de Recherche Sur Les Maladies Infectieuses Et Parasitaires (LR-MIP), Centre National de La Recherche Scientifique Et Technologique (CNRST), Bobo-Dioulasso, Burkina Faso

**Keywords:** Caffeine, Malaria infection, Insecticide resistance, Longevity, *Plasmodium falciparum*, *Anopheles coluzzi*

## Abstract

**Background:**

Alkaloids such as caffeine can be toxic for insects. However, although mosquitoes feed on many plants with nectar-containing alkaloids, the impact of these alkaloids on the vectorial capacity of mosquitoes is not known, in particular regarding increased resistance to insecticides.

**Methods:**

The effects of caffeine on *Anopheles coluzzii* female mosquitoes in terms of resistance to the insecticide deltamethrin, the rate at which mosquitoes are knocked-down during insecticide exposure, mortality within 48 h of insecticide exposure and longevity following exposure were studied. We also compared these traits for mosquitoes that were uninfected or infected by the malaria parasite *Plasmodium falciparum*. The mosquitoes were fed throughout their lives on a 10% sugar solution supplemented with 0, 50 or 200 ppm caffeine. Three or four days after emergence, they were allowed to feed on infected or uninfected blood, and 3 days later they were exposed to a deltamethrin-treated filter paper or to a sham (untreated paper). During the next 48 h the mosquitoes were checked for knockdown during the exposure and death. We measured the longevity of the surviving mosquitoes and assessed their infection status when they died.

**Results:**

The rate of mosquitoes knocked-down by the insecticide increased with higher caffeine concentrations, but neither the infection status nor its interaction with caffeine concentration influenced the knockdown rate. Caffeine also increased the mortality of the insecticide-exposed mosquitoes within 48 h after exposure. The mortality was highest if mosquitoes had fed on infected blood but harbored no parasites, and lowest if they had not fed on infected blood. In contrast, the longevity, once the mosquitoes had survived the first 48 h, was not affected by the concentration of caffeine or by its interactions with infection status or insecticide; however, the mosquitoes that had been exposed to the insecticide lived longer than unexposed ones, in particular if they had fed on infected blood but were not infected.

**Conclusions:**

Overall, the results of this experiment highlight that the level of resistance to an insecticide is affected by complex interactions between the mosquito’s diet and infection by malaria.

**Graphical Abstract:**

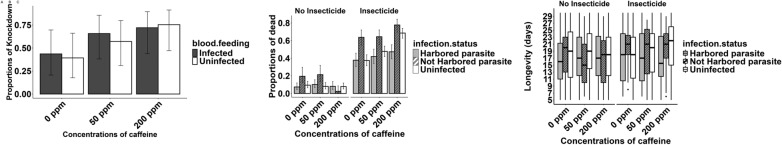

**Supplementary Information:**

The online version contains supplementary material available at 10.1186/s13071-026-07253-z.

## Background

*Anopheles gambiae* mosquitoes, the main vector of malaria in Africa, live on average less than 10 days under natural conditions [1–3] while parasites causing malaria in humans require approximately 11–14 days to complete its development into infectious stages [4, 5]. Given these life-history characteristics, most infected mosquitoes die before they are able to transmit malaria. Consequently, the transmission of malaria by parasites is highly sensitive to changes in the lifespan of mosquitoes [6–8], which is one of the major reasons why adult mosquito-targeted insecticides applied through indoor residual spraying (IRS) and insecticide-treated bed nets (ITNs) have become the cornerstone of malaria vector control measures [9, 10]. However, increasing insecticide resistance is reducing the effectiveness of these insecticides and represents a major public health threat [11–13].

Despite the growing resistance of local malaria vectors, insecticide-based vector control has significantly reduced malaria incidence across many parts of Africa, [14–16]. One explanation for the apparent discrepancy between resistance measurements and their epidemiological impact is that the effect of resistance genes is strongly influenced by environmental factors, such as food availability [17] and parasite infections [18], including malaria itself [19].

An important aspect of a mosquito’s diet is its nectar meal [20], and variations in nectar quality among plant species can influence mosquito longevity [21] and resistance to insecticides [22]. Sugars are the primary biochemical components of nectar [23], and differences in nectar composition are partly due to variations in the types and concentrations of sugars present in the nectar[24–26]. Nectar can also impact mosquitoes through its toxic compounds, such as alkaloids [27–29]. One well-known alkaloid is caffeine, which is found in the fruit and nectar of many plant species, including coffee (*Coffea spp.*), citrus, cocoa and tea plants [30–35]. Although caffeine has neurotoxic properties and can act as an insecticide [36–39], little is known about how it affects insecticide resistance in mosquitoes, either alone or in combination with malaria infection.

We therefore assessed how caffeine, at concentrations commonly found in nectar, interacts with the infectious status of mosquitoes to influence their response to insecticides. This effect of caffeine was measured using three biomarkers: the rate of knockdown during exposure, mortality within 48 h of exposure and longevity following exposure.

## Methods

The study was conducted at the Institut de Recherche en Sciences de la Santé (IRSS) de Bobo Dioulasso, in Burkina Faso. The *Anopheles coluzzii* strain from the Kou Valley (11°24′N, 4°24′59′′W) was used in all experiments. This mosquito strain exhibits metabolic resistance mediated by cytochrome P450 and target-site resistance mediated by the knockdown resistance gene (*kdr*) [40]. The mosquitoes were maintained in a biosafety room at 27 ± 2 °C, 70, 5% relative humidity under a 12:12-h light:dark (L:D) cycle.

### Experimental design

The experiment was run in three blocks. In each block, the blood of a different gametocyte carrier was used as the source of malaria parasites. Within each experimental block, we reared larvae in 40–50 trays, with each tray containing between 250 and 300 larvae in 1 L of tap water; larvae were and fed daily with Tetramin baby fish food (TetraMin Co., Melle, Germany) according to their age (day of hatching: 14 mg per tray; 1 day old: 21 mg; 2 days old: 29 mg; 3 days old: 58 mg; 4 days old: 115 mg, 5 days old or older: 216 mg). Adult males were discarded, and females were provided with continuous access to a 10% glucose solution supplemented with 0, 50 or 200 ppm of caffeine. At 3 to 4 days after emergence, females were offered the opportunity to feed on either infected or uninfected blood (see section Experimental infection for the method of infection). Each treatment was replicated in four cups of 55–65 mosquitoes each. The females that did not take a full blood meal were discarded, leaving between 18 and 30 blood-fed mosquitoes per cup. At 72 h following the blood meal—a sufficiently long time period for the blood meal to be digested—gametocytes had developed into early oocysts, and infection could be reliably confirmed they were exposed to 0.25% deltamethrin-impregnated filter paper or to a sham (untreated filter paper) for 1 h with the WHO bioassay kit [41]. The mosquitoes of each cup were tested together.

During exposure to the insecticide, we counted the number of mosquitoes that were knocked-down; at 48 h after the initial exposure we counted the number of dead mosquitoes. Although in most studies mortality within 24 h of exposure is analyzed, we chose 48 h because we observed high mortality rates during the first 2 days, followed by a substantial decrease in mortality. Consequently, we decided that an analysis based on two time periods—0 to 48 h and after 48 h—was the best choice in this experimental context. Surviving mosquitoes were maintained for at most 30 days with continual access to food, and mortality was assessed each day. Once all mosquitoes had died, the mosquitoes that had fed on infected blood were tested for the presence of malaria parasites using standard PCR methods [42]; Across the whole experiment, a total of 100 mosquitoes that had fed on uninfected blood were randomly selected and tested for malaria to ensure that no parasites were present.

### Experimental infection

*Anopheles coluzzii* female mosquitoes were fed with blood drawn from gametocyte-infected children, recruited among 5- to 12-year-old schoolchildren in Nasso village (located 13 km from Bobo-Dioulasso, Burkina Faso) using direct membrane feeding assays (DMFAs) as previously described [43–45]. Briefly, thick blood smears were collected from each volunteer, air-dried, stained with Giemsa and examined by microscopy for the presence of *Plasmodium falciparum* at the IRSS laboratory in Bobo-Dioulasso. Asexual trophozoite parasite stages were counted per 200 leucocytes, while infectious gametocyte stages were counted per 1000 leukocytes. Children with > 1000 parasites per microliter (estimated based on an average of 8000 leucocytes/ml) were treated in accordance with national guidelines. Asymptomatic *P. falciparum* gametocyte-positive children were recruited for the study.

Blood from gametocyte isolates was collected by venipuncture in heparinized tubes. Three distinct parasite isolates (named hereafter A, B and C), with gametocytemia of 56, 72 and 128 gametocytes per microliter of blood, respectively, were used for the experimental infections. DMFAs were performed by replacing donor plasma with an equivalent volume of AB+ serum from malaria-naïve European donors. After centrifugation using an Eppendorf 5702 R centrifuge (Eppendorf, Hamburg, Germany) at 37 °C at a relative centrifugal force of 11,270 *g* for 5 min, the plasma was aspirated with a pipette and replaced with AB+ serum. Blood collected from one gametocytic child was used for each block. One-half of the blood sample of each child was transferred into 1.5-ml reaction tubes and inactivated at 45 °C for 20 min at RCF = 27.17 g using a compact thermomixer [45]. The mosquitoes were starved of sugar solution for 24 h and then allowed to feed on infected or uninfected blood via membrane filters for 1 h. Female mosquitoes that did not feed or partially fed females were removed and discarded, while the remaining fully engorged mosquitoes were kept in a biosafety room under the same standard conditions (12:12-h L:D, 27 ± 2 °C, 70 ± 5% relative humidity).

### Data analysis

The statistical analyses were performed with R version R-4.4.2 [46]. We found the significance of the effects with the function Anova (package car) [47], using a type 3 SS if the interactions were significant and a type 2 SS if they were not. We analyzed the knockdown rate with a general linear model, with the function glmer, including diet, infection status of the blood meal and their interactions as fixed factors and blocks (source of blood) and cups as random effects. Since only two (0.13%) of the unexposed mosquitoes were knocked-down, we only analyzed the mosquitoes that had been exposed to the insecticide. We considered infection status of the blood meal rather than infection status of the mosquitoes as we could not determine the infection status of knocked-down mosquitoes that survived 48 h.

A general linear model was used to analyze 48-h mortality, with diet, infection status, insecticide and their interactions as fixed factors and blocks (source of blood) and cups as random effects. Infection status was classified as fed on uninfected blood, fed on infected blood and harboring parasites or fed on infected blood but not harboring any parasites.

The longevity analysis included only mosquitoes surviving the first 48 h after exposure to insecticide. Right-censoring was applied on 196 mosquitoes with a lifespan > 30 days. We used the “coxme” function that included the diet, infection status, exposure to insecticide and all interactions as fixed factors, and the block (source of blood) and cups as random factors. Infection status included the three categories mentioned above.

## Results

### Knockdown

**Fig.1:**
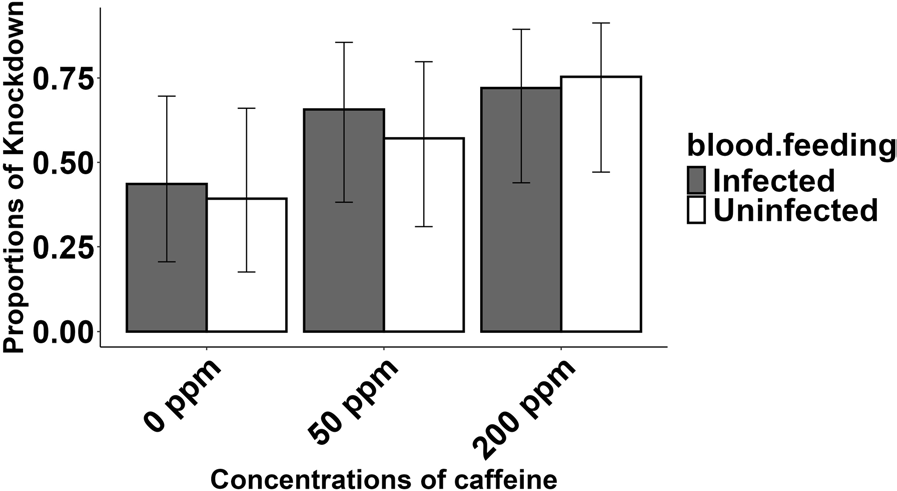
Proportion of mosquitoes knocked-down 1 h after exposure to insecticide as a function of the concentration of caffeine and their infection status. The error bars represent the 95% confidence intervals of the proportions

Of the 1632 *An. coluzzii* females exposed to insecticide, 59.6% were knocked-down within 1 h of exposure. The knockdown rate increased with increasing caffeine concentration, from 41.4% (95% confidence interval [CI]) 24.3–60.9%) at 0 ppm, 61.4% (95% CI 41.7–78.0%) at 50 ppm to 73.7% (95% CI 53.7–87.1%) at 200 ppm, (*χ*^2^ = 38.43, *df* = 1, *p* < 0.001). However, neither the type of blood ingested (*χ*^2^ = 0.55, *df* = 1, *p* = 0.459) nor the interaction between caffeine concentration and the type of blood ingested (*χ*^2^ = 1.80, *df* = 1, *p* = 0.408) had an effect on the rate of knockdown **(**Fig. 1**).**

### Mortality

Of the 1559 mosquitoes exposed to the insecticide, 53.2% (95% CI 50.7–55.6%) died within 48 h; in comparison, of the 1471 mosquitoes exposed to the sham, 9.4% (95% CI 8.0–11.0%) died. Insecticide exposure significantly increased 48-h post-exposure mortality (*χ*^2^ = 260.79, *df* = 1, *p* < 0.001).

Caffeine in the diet did not influence the mortality rate (*χ*^2^ = 0.81, *df* = 2, *p* = 0.668), but the interaction between caffeine and insecticide exposure did. In the absence of insecticide, mortality ranged from 7.0% (95% CI 5.1–9.7%) at 200 ppm caffeine to 10.2% (95% CI 7.9–13.1%) at 0 ppm caffeine, whereas in the presence of insecticide, mortality was 43.6% (95% CI 39.3%–48.0%) at 0 ppm caffeine, 50.4% (95% CI 46.1–54.6%) at 50 ppm caffeine and 64.8% (95% CI 60.7–68.7%) at 200 ppm caffeine (*χ*^2^ = 17.76, *df* = 2, *p* < 0.001).

Mortality was also affected by infection status. The mortality rate was 23.9% (95% CI 21.3–26.7%) among those mosquitoes harboring parasites, and 47.4% (95% CI 43.4–51.4%) among mosquitoes that did not harbor any parasites; uninfected mosquitoes had a mortality rate of 30.8% (95% CI 28.5–33.2%) (*χ*^2^ = 17.50, *df* = 2, *p* < 0.001).

There was a significant interaction between insecticide exposure and infection status (*χ*^2^ = 7.25, *df* = 2, *p* = 0.026). In mosquitoes harboring parasites, exposure to the insecticide increased mortality from 8.5% (95% CI 6.4–11.3%) to 42.3% (95% CI 37.7–46.9%); in those not harboring parasites, mortality increased from 14.2% (95% CI 10.3–19.2%) to 68.6% (95% CI 63.7–73.1%); and in uninfected mosquitoes, mortality increased from 8.4% (95% CI 6.6–10.7%) to 51.9% (95% CI 48.4–55.5%).

**Fig. 2:**
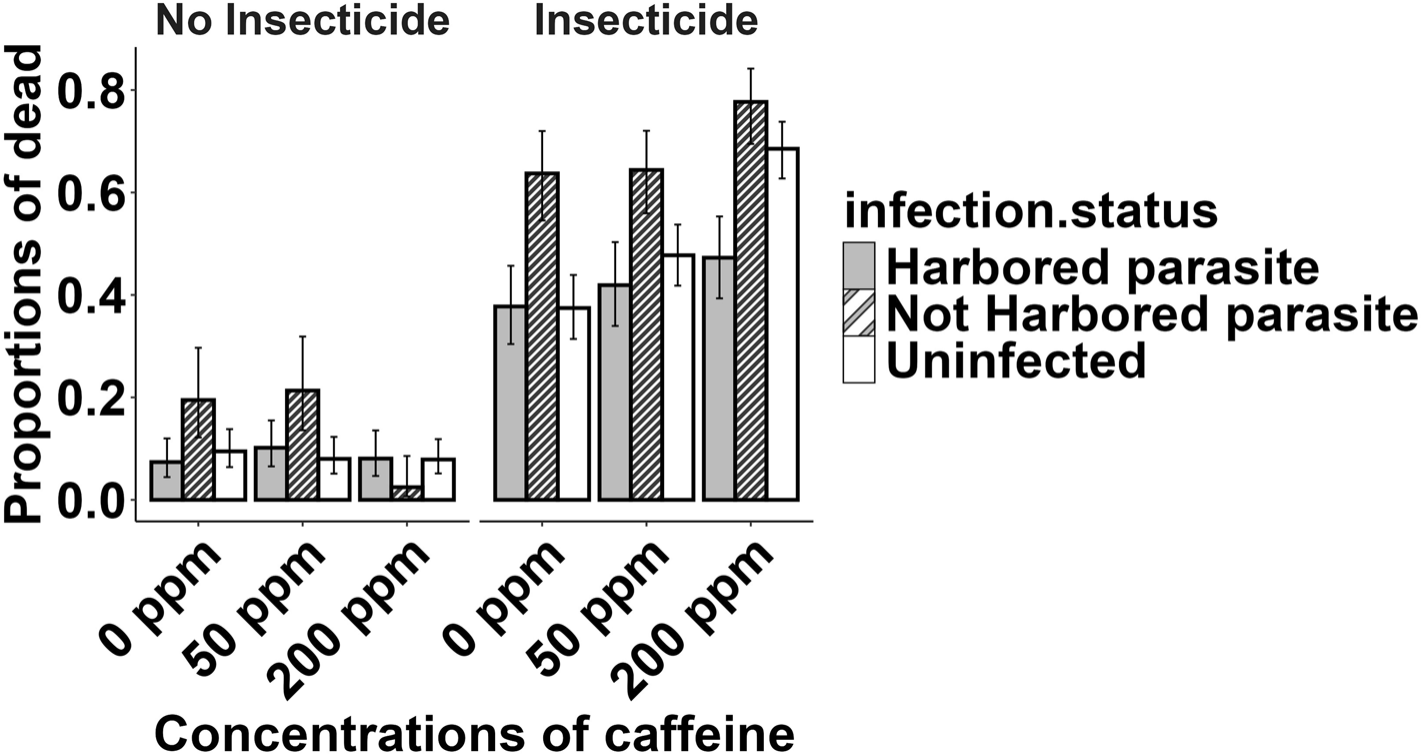
Mortality within 48 h of exposure as a function of the exposure to the insecticide, concentration of caffeine and the infection status of mosquitoes. The error bars represent the 95% confidence intervals of the proportions

Mortality tended to increase more with caffeine concentration in uninfected mosquitoes than in infected mosquitoes, although this interaction was not quite significant (*χ*^2^ = 8.87, *df* = 4, *p* = 0.064). In contrast, a significant three-way interaction between infection status, insecticide exposure and caffeine concentration did influence mosquito mortality (*χ*^2^ = 9.85, *df* = 4, *p* = 0.043). For example, at 0 ppm caffeine, mortality after insecticide exposure was highest for those mosquitoes that did not harbor parasites (63.7%, 95% CI 54.5–72.0%), compared with mortality in infected (37.7%, 95% CI 30.4–45.7%) and uninfected individuals (37.4%, 95% CI 31.4–43.9%). At 200 ppm caffeine, mortality in non-exposed mosquitoes without parasites was low (2.5%, 95% CI 0.7–8.6%); however, those mosquitoes which were exposed to insecticide showed the highest mortality (77.7%, 95% CI 69.5–84.2%), compared with uninfected (68.5%, 95% CI 62.7–73.8%) and infected (47.3%, 95% CI 39.3–55.3%) mosquitoes **(**Fig. 2**)**.

### Longevity

**Fig. 3:**
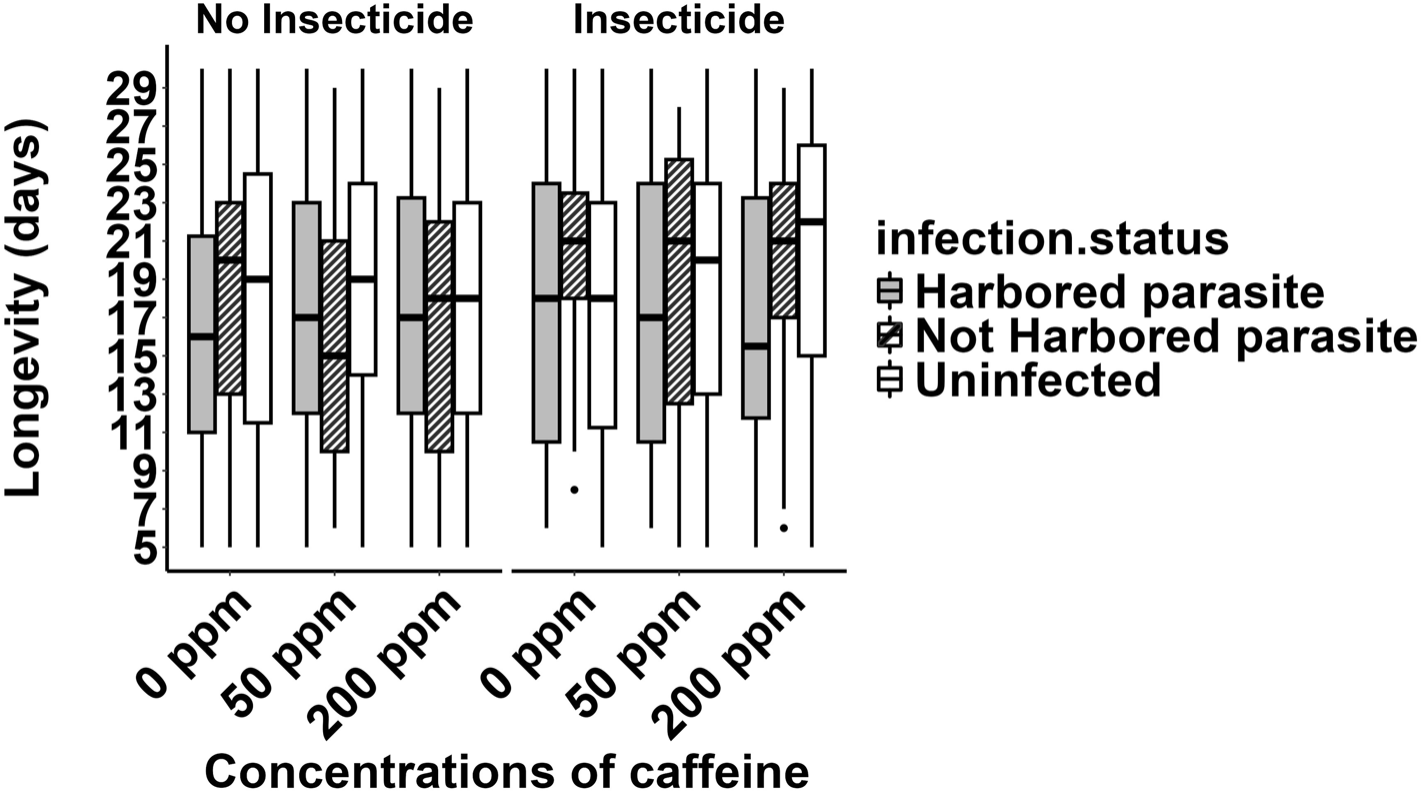
Longevity starting from 48 h after exposure as a function of the exposure to the insecticide, effects of different concentrations of caffeine and the infection status of mosquitoes. The rectangles represent the longevities between the 25th and the 75th percentiles, the horizontal lines within the rectangles denote the medians. The vertical lines span 1.5-fold above the 75th percentiles and below 25th percentiles, and the dots show outliers that are beyond this range

The average longevity of the 2063 mosquitoes that survived the first 48 h was 18.6 days, and it was not significantly affected by caffeine concentration (*χ*^2^ = 1.46, *df* = 2, *p* = 0.48) or by any of its interactions (*χ*^2^ =  6.4, *df* = 2.4, *p* > 0.17) **(**Fig. 3**).**

Longevity was about 1 day longer among insecticide-exposed mosquitoes than among unexposed ones (*χ*^2^ = 4.49, *df* = 1, *p* = 0.034). The effect of exposure depended on whether the mosquitoes were infected by malaria. In mosquitoes harboring parasites, the average longevity was 18.3 (95% CI 17.5–19.1) days without exposure to the insecticide and 17.7 (95% CI 16.5–18.8) days with exposure. In mosquitoes that did not harbor parasites, the average longevity was 17.5 days (95% CI 16.3–18.6) without insecticide exposure and 19.6 days (95% CI 17.9–21.9) with exposure. In uninfected mosquitoes, the average longevity was 20.0 (95% CI 19.0–20.9) days with insecticide exposure and 18.7 (95% CI 18.0–19.3) days without exposure (interaction exposure × infection status: *χ*^2^ = 6.06, *df* = 2,* p* = 0.048) (Fig. 3).

## Discussion

Our study examined how caffeine and mosquito infection status influence insecticide resistance phenotype and the longevity of surviving mosquitoes after exposure.

We found that increasing caffeine concentration increased the mortality of *An. coluzzii* females after insecticide exposure. Caffeine, an alkaloid, may enhance insecticide efficacy in *An. gambiae* by acting as a neurotoxin and inducing oxidative stress [48–50]. Insecticide exposure itself also triggers reactive oxygen species (ROS) production via cytochrome P450 activity, and is associated with elevated levels of antioxidant enzymes, such as glutathione, peroxidase and catalase [19, 51, 52]. We speculate that the combination of insecticide detoxification and caffeine-induced oxidative stress can overwhelm physiological defenses, impair detoxification enzyme efficiency, reduce resistance and, ultimately, increase mortality.

Our findings align with those of Cissé-Niambélé et Koella (unpublished) that show increased mortality of *An. coluzzii* females with increasing caffeine concentrations. Studies on *Aedes* larvae have shown a similar dose-dependent toxicity of caffeine [53, 54]. The decrease in resistance at higher caffeine concentrations may also be explained by a repellent effect of the compound [55], which leads to smaller blood meals and lower energy reserves. As caffeine concentration increases, this repellent effect may become stronger, reducing meal intake and making mosquitoes more susceptible to insecticide exposure.

Infected mosquitoes that did not ultimately harbor detectable parasites may have mounted an immune response against the infection [56, 56, 57]. This immune activation is often associated with the increased production of ROS [58, 58, 59, 59], which can amplify the cytotoxic effects of insecticides and contribute to higher mortality [60]. Therefore, the elevated mortality rate may result from the combined effects of the energetic cost of the immune response and the oxidative stress induced by the insecticide toxicity.

The absence of an effect of caffeine on mosquito longevity was unexpected, given that caffeine is both a neurotoxic and pro-oxidant, and particularly since it significantly affected mosquito resistance within 48 h of insecticide exposure. This result contrasts with results from several previous studies reporting on the effects of caffeine on various mosquito life-history traits. For example, caffeine has been shown to increase larval locomotion [61] and, conversely, to reduce fertility in *Aedes albopictus* [62]. Other studies have reported that a caffeine-based diet reduces the longevity of *Aedes albopictus* at concentrations similar to those used in our study [55]. Similarly, reduced longevity has been observed in fly species such as *Musca domestica* [63], *Drosophila prosaltans* and *D. melanogaster* [64, 65] following caffeine consumption. Interestingly, caffeine has been reported to increase the lifespan of bees [66], highlighting species-specific responses to the compound.

The improved survival of mosquitoes exposed to insecticide may be attributed to selective sorting that favors larger individuals, which likely benefit from energy reserves accumulated during the larval stage [17, 67]. Larger mosquitoes tend to have higher general reserves, which can enhance their longevity [68]. This phenomenon could be explained by the fact that exposure to gametocytes, even without establishing an infection, may trigger an immune or metabolic response, enhancing long-term survival after the elimination of the weakest individuals [45, 69].

Our study has several limitations. First, the mosquitoes used originated from a single laboratory strain with a high level of insecticide resistance and were reared under controlled conditions, which may limit the generalizability of our findings to wild *An. gambiae* populations with different ecological and physiological characteristics. Second, the detection of *Plasmodium* DNA in some mosquitoes does not confirm the presence of viable or developing parasites, as PCR can amplify DNA from both viable or non-viable parasites. Finally, since only one insecticide (deltamethrin) was tested, the results cannot be extrapolated to other insecticide classes, to which resistant mosquitoes may respond differently.

## Conclusions

Our results highlight the potential of caffeine as a complementary vector control tool that is capable of enhancing mosquito susceptibility to insecticides. Caffeine could be incorporated into attractive targeted sugar baits to improve their effectiveness. Overall, this study underscores the complex relationship between diet, insecticide resistance phenotype and infection status of mosquitoes.

## Supplementary Information

**Additional file 1.** Database of mortality and longecity data.

**Additional file 2.** Script for statictical analyses for Knockdown.

**Additional file 3.** Database of knockdown data.

**Additional file 4. ** Script for statidtical analyses of mortality and longevity data.

## Data Availability

Data supporting the main conclusions of this study are included in the manuscript.
